# Prevalence and Associated Factors for HPV in People Living with HIV: Are INSTIs Protective Against HPV-16? The GAIA Study

**DOI:** 10.3390/v17081147

**Published:** 2025-08-21

**Authors:** Omar Hernández-López, Brenda Clara González-Contreras, Ana Luz Cano-Díaz, José Antonio Mata-Marín, Ericka Nelly Pompa-Mera, Javier Vicente Noyola-Gómez, Salma Triana-González, Paola Edith Padilla-Noguera, Alberto Chaparro-Sánchez, Sócrates Alberto García-Gutiérrez, Gustavo Barriga-Angulo, Jesús Enrique Gaytan-Martinez

**Affiliations:** 1 Infectious Diseases Department, Hospital de Infectología “La Raza”, National Medical Center, Instituto Mexicano del Seguro Social, Mexico City 02990, Mexico; omar291193@gmail.com (O.H.-L.); ana.knodiaz@gmail.com (A.L.C.-D.); stg.9204@gmail.com (S.T.-G.); a_chaparro@hotmail.com (A.C.-S.); jgaytanmtz@yahoo.com.mx (J.E.G.-M.); 2 Medicine School, Universidad Autónoma Metropolitana, Mexico City 04960, Mexico; bcgc150901@gmail.com; 3 Research Unit, Hospital de Infectología “La Raza”, National Medical Center, Instituto Mexicano del Seguro Social, Mexico City 02990, Mexico; erickanelly@yahoo.com.mx; 4 Gynecology Department, Hospital de Infectología “La Raza”, National Medical Center, Instituto Mexicano del Seguro Social, Mexico City 02990, Mexico; gyneconoyola@gmail.com; 5 Medicine School, Facultad de Estudios Superiores Iztacala, Universidad Nacional Autónoma de México, Mexico City 54090, Mexico; paolaedith1b@gmail.com; 6 Pathology Department, Hospital de Especialidades “Dr. Antonio Fraga Mouret”, “La Raza” National Medical Center, Instituto Mexicano del Seguro Social, Mexico City 02990, Mexico; sagg_888@hotmail.com; 7 Laboratory Department, Hospital de Infectología “La Raza”, National Medical Center, Instituto Mexicano del Seguro Social, Mexico City 02990, Mexico; gustavo.barriga@imss.gob.mx

**Keywords:** human papillomavirus, HIV, INSTI, anal cancer, antiretroviral therapy, HPV-16

## Abstract

Human papillomavirus (HPV) significantly contributes to anogenital cancers, with elevated risks among people living with HIV (PWH), particularly men who have sex with men (MSM). This study assessed anal HPV prevalence and associated risk factors in PWH in Mexico, focusing on the role of antiretroviral therapy (ART). **Methods:** A cross-sectional study at an HIV clinic in Mexico City (October 2023–December 2024) enrolled 214 MSM with HIV. The participants completed a validated risk factor questionnaire and provided anal samples for real-time PCR testing of 28 HPV genotypes. Logistic regression analyzed associations between HPV infection, ART regimens, and clinical/behavioral factors. **Results:** HPV prevalence was 89.3%, with HPV-16 (20.1%) being the most common high-risk genotype. Integrase inhibitor (INSTI) use was inversely associated with HPV-16 infection (OR: 0.42; 95% CI: 0.21–0.83; *p* = 0.011), while protease inhibitor use increased HPV-16 (OR: 2.16; 95% CI: 1.09–4.29; *p* = 0.025) and HPV-6 risks. Higher CD4+ counts (≥500 cells/mm^3^) and undetectable HIV viral load (<40 copies/mL) were protective against multiple HPV genotypes. Lower education and smoking increased HPV risk. **Conclusions:** This first Mexican study in the ART and HPV vaccination era highlights high anal HPV prevalence in PWH and suggests that INSTI-based regimens may reduce HPV-16 risk, informing ART selection for HPV prevention.

## 1. Introduction

Human papillomavirus (HPV) is the primary cause of nearly all cervical cancers and significantly contributes to other anogenital and oropharyngeal cancers [1]. Anal cancer, a malignancy representing 2.7% of gastrointestinal tract tumors, led to 1670 deaths in the USA in 2022, with approximately twice as many new cases in females compared to males [2]. As the most common sexually transmissible infection worldwide, HPV has a profound negative impact on individual’s social lives [3]. The highest excess cancer incidence and mortality in people living with HIV (PWH) compared to the general population is observed for HPV-related cancers [4]. High-risk HPV (HR-HPV) subtypes, including HPV 16, 18, 31, 33, 35, 39, 45, 51, 52, 56, 58, and 59, can cause intraepithelial lesions and various HPV-related cancers, such as cervical, oropharyngeal, penile, and anal cancers, with HPV 16 and 18 being the most carcinogenic [5]. While HPV infections are typically cleared by the host immune system within 1–2 years [6], PWH, particularly those with immune dysfunction, have an impaired ability to clear the virus [7]. Men who have sex with men (MSM), especially those who are HIV-positive, face a significantly higher risk of anal cancer [8]. HIV-positive MSM have the highest relative risk, with a 37-fold increase for anal cancer, followed by solid organ transplant recipients (10-fold increased risk), MSM without specified HIV status (17-fold increased risk), individuals with inflammatory bowel disease (2–3-fold increased risk), and smokers (2–4-fold increased risk) [9]. Anal HPV infections in PWH exhibit lower clearance rates and longer persistence [10], with multiple genotypes detected in over 80% of cases [11]. HPV vaccination is effective in preventing HPV-related diseases, including anal cancer, and an increasing number of countries are implementing vaccination programs targeting men or MSM [12]. Vaccination in HPV-positive patients may enhance HPV remission in cervical swabs, making it a valuable component of secondary prevention [13]. Currently, no studies in Mexico have evaluated the factors associated with HPV prevalence in the era of widespread HPV vaccination or the relationship between antiretroviral therapy (ART) and high-risk HPV in the anal region among PWH

## 2. Materials and Methods

### 2.1. Study Design

A cross-sectional study was conducted at the HIV Clinic of the Hospital de Infectología, National Medical Center “La Raza,” Mexico City, Mexico, from October 2023 to December 2024.

### 2.2. Study Population

The participants were recruited from the HIV Clinic based on specific inclusion and exclusion criteria. Eligible participants included men aged 18 years or older who self-identified as men who have sex with men (MSM) engaging in receptive anal sex, were willing to complete a risk factor questionnaire for HPV infection, and consented to provide an anal brushing sample for HPV polymerase chain reaction (PCR) testing. The exclusion criteria included men exclusively practicing insertive anal sex, refusal to complete the risk factor questionnaire, or declining to provide an anal brushing sample for HPV PCR testing. All eligible participants were informed about the study’s objectives, procedures, and their rights and provided written informed consent prior to enrollment.

### 2.3. Measurements

The participants completed a validated 39-item questionnaire to assess risk factors for anal HPV infection. The questionnaire was administered by trained personnel in a private setting to ensure confidentiality and promote accurate reporting. It collected the following data:

- Sociodemographic characteristics: age, education level, occupation, and socioeconomic status.
- HIV-related parameters: baseline CD4+ cell count and HIV-RNA viral load (measured via PCR).
- Sexual practices: number of sexual partners, frequency of receptive anal sex, condom use, and history of sexually transmitted infections (STIs).
- HPV vaccination status: prior receipt of HPV vaccination and type of vaccine administered.

Anal brushing samples were collected following a detailed explanation of the procedure to the participants. A non-sterile endocervical brush was gently inserted 5 mm into the anal canal and rotated to collect epithelial cells. The brush was then placed in a disposable virus transport tube containing a preservation solution composed of Hank’s balanced salt solution supplemented with antibiotics, antifungals, and a stabilizing agent to maintain viral DNA integrity. The samples were stored at 4 °C and transported to the laboratory within 2 h for processing.

The anal brushing samples were analyzed using real-time PCR to detect HPV DNA, targeting 28 HPV genotypes, including the following:

- High-risk genotypes: 16, 18, 26, 31, 33, 35, 39, 45, 51, 52, 53, 56, 58, 59, 66, 68, 69, 73, 82.
- Low-risk genotypes: 6, 11, 40, 42, 43, 44, 54, 61, 70.

The PCR assay followed standardized protocols, incorporating positive and negative controls to ensure accuracy.

The results were reported as positive or negative for each genotype and categorized into high-risk and low-risk HPV types.

### 2.4. Statistical Analysis

The data were analyzed using descriptive and inferential statistical methods. Categorical variables, such as sociodemographic characteristics and HPV prevalence, were summarized using frequencies, proportions, and percentages. Continuous variables, such as CD4+ cell count, are described using means and standard deviations or medians and interquartile ranges, based on data distribution.

Associations between relevant factors and high-risk HPV infection were evaluated using chi-square tests or Fisher’s exact tests for categorical variables. A bivariate analysis was performed with binary logistic regression to assess the independence of the associated factors expressed in odds ratios (ORs) with 95% confidence intervals (CIs). A multivariate analysis was performed using a regression model including only variables with *p* < 0.05 from the bivariate analysis. The variables included in the multivariate model were the following: INSTI use, PI use, HIV-1 RNA < 40 copies/mL (undetectable), INSTI use for more than 12 months, CD4+ ≥ 500 cells/mm^3^, CD4+ ≥ 200 cells/mm^3^, HIV-1 RNA < 40 copies/mL, smoking, consistent condom use, higher educational level, and lower educational level. All statistical analyses were performed using SPSS version 26.0 (IBM Corp., Armonk, NY, USA). Data with *p* < 0.05 were considered statistically significant.

### 2.5. Ethical Considerations

The study was approved by the Institutional Review Board of the National Medical Center “La Raza,” Health Research Committee 3502 (protocol number: R-2025-3502-052). All procedures complied with the ethical principles outlined in the Declaration of Helsinki. Participants’ confidentiality was ensured through anonymized data and secure storage of personal information.

## 3. Results

### 3.1. Study Population and Baseline Characteristics

Between October 2023 and December 2024, 214 PWH were enrolled. The median age was 30 years (IQR 25–36), with 185 (86.4%) participants identifying as Hispanic, and 39.3% (*n* = 84) of them holding a bachelor’s degree. Most participants (200, 93.5%), had a prior HIV diagnosis, with a median time since diagnosis of 18 months (IQR 12–29), while 14 (6.5%) were newly diagnosed. Immunovirological outcomes included a median CD4+ cell count of 625 cells/mm^3^ (IQR 431–879), with 187 (87.4%) subjects achieving an HIV-1 RNA viral load < 40 copies/mL. Among those with a prior HIV diagnosis, the median duration of ART was 12 months (IQR 6–24). Of these, 126 (58.9%) received INSTI-based regimens, and 32.2% (*n* = 69) were on PI-based regimens. See Table 1 for detailed baseline characteristics.

### 3.2. Behavioral Factors Associated with HPV Infection

The median age at first sexual intercourse was 17 years (IQR 15–19), and the median age at first receptive anal intercourse was 18 years (IQR 16–21). Over half of the participants (120, 56.1%) reported 1–4 sexual partners per year, and 157 (73.4%) reported 0–50 instances having engaged in receptive anal sex. Consistent condom use was reported by 182 (85.0%) participants, while 28 (13.1%) reported anal object insertion. Regarding coinfections, 55 (25.7%) subjects had a history of syphilis; in addition, 57 (26.6%) were smokers, and 184 (86.0%) reported alcohol consumption in the past 12 months. Most participants (155, 72.4%) were not vaccinated against HPV. See Table 1 for detailed baseline characteristics.

### 3.3. HPV Infection Prevalence

**Figure 1:**
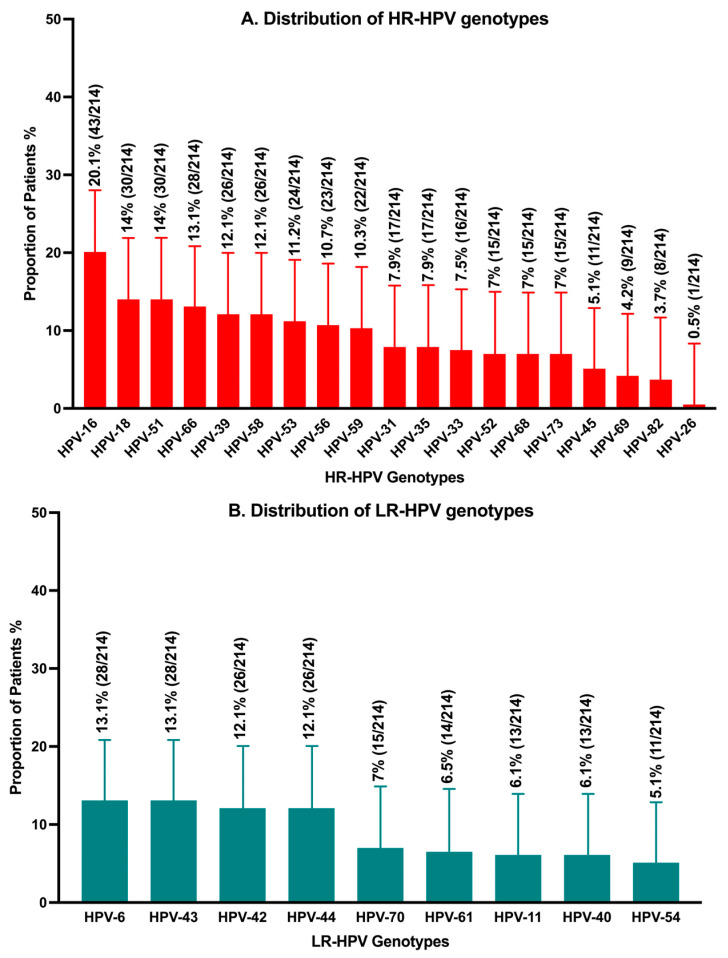
Prevalence of high- and-low risk HPV genotypes (*n* = 214): (**A**). Distribution of high-risk human papillomavirus genotypes. (**B**). Distribution of low-risk human papillomavirus genotypes.

At least one HPV genotype was detected in 191 (89.3%) participants. Half (50.5%, *n* = 108) of them had both high-risk and low-risk genotypes, and 144 (67.3%) had two or more genotypes. The most common high-risk genotypes were HPV-16 (20.1%, *n* = 43), followed by HPV-18 and HPV-51 (both 14.0%, *n* = 30). The most frequent low-risk genotypes were HPV-6 and HPV-43 (both 13.1%, *n* = 28). See Figure 1 for HPV genotype prevalence.

### 3.4. Factors Associated with HPV Infection

Significant associations were identified between ART regimens and HPV genotypes. INSTI use was inversely associated with HPV-16 infection (OR: 0.42; 95% CI: 0.21–0.83; *p* = 0.011). Conversely, PI use was associated with increased risk of HPV-16 (OR: 2.16; 95% CI: 1.09–4.29; *p* = 0.025) and HPV-6 (OR: 2.38; 95% CI: 1.06–5.32; *p* = 0.031) infection. ART use, regardless of the regimen, was inversely associated with HPV-39 (OR: 0.18; 95% CI: 0.05–0.62; *p* = 0.012), HPV-66 (OR: 0.20; 95% CI: 0.06–0.68; *p* = 0.016), and HPV-40 (OR: 0.17; 95% CI: 0.04–0.73; *p* = 0.035) infections. In the multivariate analysis, INSTI use was independently associated with a protective effect against HPV-16 infection (aOR: 0.43; 95% CI 0.21–0.86; *p* = 0.018). Significant protective associations were also observed for INSTI use with HPV-31 (aOR: 0.35; 95% CI: 0.16–0.78; *p* = 0.008), HPV-33 (aOR: 0.018; 95% CI: 0.001–0.327; *p* = 0.007), HPV-39 (aOR: 0.21; 95% CI: 0.06–0.70; *p* = 0.011), and HPV-66 (aOR: 0.24; 95% CI: 0.07–0.82; *p* = 0.023) infections. Similarly, protease inhibitor (PI) use demonstrated a protective trend against HPV-33 (aOR: 0.047; 95% CI: 0.003–0.836; *p* = 0.037), HPV-39 (aOR: 0.22; 95% CI: 0.06–0.81; *p* = 0.022), and HPV-66 (aOR: 0.24; 95% CI: 0.06–0.93; *p* = 0.038). In contrast, INSTI use was associated with an increased risk of HPV-6 infection (aOR: 2.53; 95% CI: 1.09–5.85; *p* = 0.030).

### 3.5. Immunovirological Factors Also Showed Associations

A CD4+ cell count > 500 was inversely associated with high-risk genotype (OR: 0.48; 95% CI: 0.27–0.86; *p* = 0.013) and low-risk genotype (OR: 0.45; 95% CI: 0.24–0.83; *p* = 0.010) infections. A CD4+ cell count > 200 cells/mm^3^ was inversely associated with HPV-82 infection (OR: 0.10; 95% CI: 0.01–0.61; *p* = 0.039). An undetectable HIV-1 RNA viral load (<40 copies/mL) was inversely associated with infections by high-risk genotypes (OR: 0.40; 95% CI: 0.15–0.95; *p* = 0.049) and HPV-82 (OR: 0.10; 95% CI: 0.01–0.61; *p* = 0.039), HPV-45 (OR: 0.22; 95% CI: 0.06–0.82; *p* = 0.036), HPV-53 (OR: 0.22; 95% CI: 0.08–0.58; *p* = 0.001), and HPV-44 (OR: 0.32; 95% CI: 0.12–0.86; *p* = 0.019). In the multivariate analysis, having an undetectable HIV-1 RNA viral load (<40 copies/mL) was independently associated with significantly lower odds of HPV-53 infection (aOR: 0.172; 95% CI: 0.05–0.50; *p* < 0.001). A protective association remained statistically significant with CD4+ > 200 cells/mm^3^ for HPV-82 (aOR: 0.067; 95% CI: 0.008–0.56; *p* < 0.013).

### 3.6. Educational Attainment Influenced HPV Infection Risk

Having a lower educational level (high school or less) was associated with increased risk of HPV-16 (OR: 2.15; 95% CI: 1.09–4.23; *p* = 0.025), HPV-59 (OR: 4.12; 95% CI: 1.41–12.01; *p* = 0.006), and HPV-53 (OR: 2.75; 95% CI: 1.16–6.54; *p* = 0.018) infections. In the multivariate analysis, a lower educational level was independently associated with an increased risk of HPV-16 acquisition (aOR: 2.05; 95% CI: 1.03–4.08; *p* = 0.040), while having a higher educational level was independently associated with protection against HPV-53 infection (aOR: 0.176; 95% CI: 0.48–0.65; *p* = 0.009).

### 3.7. Behavioral Factors Also Showed Associations

Consistent condom use was inversely associated with HPV-39 infection (OR: 0.84; 95% CI: 0.79–0.89; *p* = 0.030). Smoking was associated with increased risk of HPV-53 (OR: 2.64; 95% CI: 1.11–6.31; *p* = 0.024) and HPV-44 (OR: 2.72; 95% CI: 1.17–6.31; *p* = 0.016) infections. Alcohol consumption and HPV vaccination status showed no significant associations with HPV infection. In the multivariate analysis, smoking was independently associated with increased odds of HPV-53 infection (aOR: 3.025; 95% CI: 1.16–7.88; *p* = 0.024); HPV-44 (aOR: 2.619; 95% CI: 1.087–6.313; *p* = 0.032). In contrast, consistent condom use was significantly associated with a protective effect against HPV-31 infection (aOR: 0.292; 95% CI: 0.091–0.932; *p* = 0.038). Refer to Table 2 for the bivariate analysis and to Table 3 for the multivariate analysis using logistic regression. These analyses were conducted for all detected HPV genotypes. The multivariate model only included variables with *p* < 0.05 from the bivariate analysis. The full results of the multivariate model, including all variables regardless of statistical significance, are available in Supplementary Table S1.

## 4. Discussion

In this cross-sectional study involving 214 PWH, a high prevalence of HPV infection was observed, with at least one genotype detected in 89.3% of the participants. The use of INSTIs was inversely associated with HPV-16 infection, suggesting a potential protective effect. Conversely, PI use was associated with an increased risk of HPV-16 and HPV-6 infections.

Several immunovirological and behavioral factors were significantly associated with HPV infection. A CD4+ cell count ≥ 500 cells/mm^3^ was linked to a lower risk of infection with both high- and low-risk HPV genotypes. An undetectable viral load was associated with a reduced prevalence of HPV-45, HPV-53, and HPV-44. Consistent condom use was correlated with a decreased risk of HPV-39 infection, while smoking was associated with an increased risk of HPV-53 and HPV-44 infections. Regarding educational attainment, a high school or lower education level was associated with a higher risk of HPV-16, HPV-59, and HPV-53 infections, whereas a university-level education was linked to a reduced risk of HPV-53 infection. These findings highlight the interplay of immunological, therapeutic, and behavioral factors in the prevalence of HPV infection among PWH, emphasizing the need for comprehensive strategies for prevention and management.

The observed prevalence of anal HPV infection (89.3%) aligns with the global estimates for PWH, whose prevalence rates often exceed 80% due to impaired immune clearance mechanisms because the incidence of anal cancer is 30 times higher in this population [14,15]. The high burden of multiple HPV genotypes (67.3%) and the predominance of high-risk genotypes such as HPV-16 (20.1%), HPV-18 (14.0%), and HPV-51 (14.0%) underscore the elevated risk of HPV-related cancers, particularly anal cancer, in this population. These findings are consistent with prior studies reporting HPV-16 and HPV-18 as the most carcinogenic genotypes, contributing significantly to anal intraepithelial neoplasia and malignancy [16].

A notable finding was the association between ART regimens and HPV infection risk. The inverse association between INSTI-based regimens and HPV-16 infection suggests a potential protective effect, which is a novel observation in the context of anal HPV infection among PWH. This may be attributed to the favorable immunological profile of INSTI-based regimens, which are known to promote rapid CD4+ T-cell recovery and viral suppression, potentially enhancing HPV clearance [17]. The potential protective association between the use of INSTIs and HPV-16 infection could be attributed to the favorable profile of these antiretrovirals, promoting mucosal immune restoration [18]. INSTIs induce rapid viral suppression and exhibit a reduced inflammatory impact, which may facilitate an enhanced local immune response in anogenital mucosae, thereby promoting viral clearance or preventing HPV persistence [19,20].

Conversely, the use of PIs was associated with an increased risk of HPV infections. PIs may induce damage to the genital and anal mucosae and increased endothelial damage, which could favor HPV persistence. This damage may be related to PI-associated side effects, such as lipodystrophy, which compromise mucosal integrity and heighten the susceptibility to infections [21,22,23]. This finding could reflect differences in immune restoration or potential interactions between PIs and host immune responses to HPV, though the underlying mechanisms remain unclear. Previous studies have suggested that PIs may alter immune cell function or cytokine profiles, potentially impacting HPV persistence [24,25]. Interestingly, this association contrasts with our findings for HPV-16, the most oncogenic genotype, which showed increased risk of HPV-16 infection with PI use; however, a protective effect was observed for other high-risk genotypes such as HPV-33, HPV-39, and HPV-66. This information should be taken with caution, since only 16 patients had genotype HPV-33, 24 had genotype HPV-39, and 28 had genotype HPV-66. Further studies with a larger sample size should be conducted. Caputo suggested that is possible that PI-based therapy may contribute to mucosal immune recovery by restoring the local immune balance and enhancing the expression of innate defense molecules such as SLPI and defensins [26]. In light of these discrepancies, the precise role of PIs in HPV pathogenesis remains unclear, and further prospective studies are warranted to elucidate their impact. The protective effect of ART, regardless of the regimen, against HPV-39, HPV-66, and HPV-40 infections further supports the role of immune restoration in reducing HPV prevalence, as ART-mediated viral suppression and CD4+ recovery likely enhance mucosal immunity [27]. Immunovirological factors were also significant predictors of HPV infection. A CD4+ cell count ≥ 500 cells/mm^3^ was inversely associated with both high-risk and low-risk HPV genotypes, consistent with the established link between immune competence and HPV clearance [28].

Similarly, an undetectable HIV-1 RNA viral load (<40 copies/mL) was associated with a lower prevalence of high-risk genotypes and specific genotypes such as HPV-45, HPV-53, and HPV-44. These findings highlight the importance of sustained virological control in mitigating HPV infection risk, likely due to improved immune surveillance and reduced inflammation in the anal mucosa [29]. The specific association of a CD4+ count > 200 cells/mm^3^ with reduced HPV-82 infection suggests that even a moderate immune recovery can confer protection against certain genotypes, though the clinical significance of HPV-82 remains less well characterized compared to that of HPV-16 and HPV-18 [30].

Behavioral factors played a critical role in HPV infection risk. Consistent condom use was inversely associated with HPV-31 and HPV-39 infections, reinforcing the protective effect of barrier methods against HPV transmission, as previously reported [31]. However, the high prevalence of HPV, despite 85% of the participants reporting consistent condom use, suggests that condoms may not fully prevent HPV transmission, particularly in the context of receptive anal sex, where skin-to-skin contact in the perianal region remains a risk [32].

Smoking was associated with an increased risk of HPV-53 and HPV-44 infections, consistent with evidence that tobacco use impairs mucosal immunity and promotes HPV persistence through oxidative stress and immune suppression [33]. The lack of association between alcohol consumption and HPV infection may reflect the moderate alcohol use reported by most participants (86%), which may not have reached the threshold for significant immune modulation [34].

Educational attainment emerged as a significant social determinant of HPV infection risk. The participants with a high school education or less had an increased risk of HPV-16, HPV-59, and HPV-53 infections, while a college education was inversely associated with HPV-53 infection. These findings suggest that higher education may correlate with greater health literacy, access to preventive measures, or healthier behaviors, such as reduced smoking or increased engagement with healthcare services [35]. This aligns with global studies showing that socioeconomic factors, including education, influence STI outcomes through access to information and resources [36].

The absence of a significant association between HPV vaccination status and HPV infection prevalence is notable, given that 72.4% of the participants were unvaccinated. This may reflect the limited implementation of HPV vaccination programs targeting MSM in Mexico, as well as the timing of vaccination relative to HPV exposure, which can reduce vaccine efficacy [37]. The high prevalence of HPV infection despite vaccination in some participants suggests that vaccination may have occurred after HPV exposure, limiting its protective effect. Future studies should explore the impact of early vaccination in MSM and PWH, particularly in settings with emerging vaccination programs.

This study provides the first comprehensive analysis of anal HPV infection prevalence and associated risk factors among PWH in Mexico during the era of HPV vaccination and modern ART. The high prevalence of HPV and multiple genotypes highlights the urgent need for targeted screening and prevention strategies in this population. Notably, the study identifies a novel association between INSTI-based regimens and reduced HPV-16 infection risk, in contrast with an increased risk of specific genotypes linked to PI-based regimens. Specifically, PIs appear to increase the risk of HPV-16, the genotype most associated with malignant neoplasms, while offering a protective effect against other high-risk genotypes, such as HPV-33, HPV-39, and HPV-66. These findings necessitate further research to clarify the underlying immunological or pharmacological mechanisms. Moreover, integrating HPV vaccination, smoking cessation programs, and consistent condom use into comprehensive HIV care is critical to reducing the burden of HPV-related diseases.

This study utilized a robust real-time PCR assay targeting 28 HPV genotypes, ensuring the accurate detection and differentiation of high- and low-risk genotypes. By including a diverse set of sociodemographic, immunovirological, and behavioral variables, the study thoroughly evaluated risk factors, revealing significant associations with ART regimens, CD4+ cell counts, and viral load suppression. Focusing on MSM, a group at high risk for anal HPV infection and related cancers, enhances the study’s relevance for targeted public health interventions.

However, the cross-sectional design limited the ability to establish causality or assess the temporal dynamics of HPV infection and clearance in PWH. The study population, restricted to MSM attending a single HIV clinic in Mexico City, may not fully represent other PWH populations in Mexico or globally, which potentially limits the findings’ generalizability. Self-reported behavioral data, such as condom use and sexual practices, may be subject to recall or social desirability bias, despite efforts to ensure confidentiality. Additionally, the limited sample size reduced the statistical power but provides a foundation for future research and raises critical questions for further investigation.

These findings underscore the need for longitudinal studies to explore causal relationships between ART regimens, immune status, and HPV infection outcomes in PWH. Future research should investigate the potential protective mechanisms of INSTI-based regimens against specific HPV genotypes and clarify the impact of PI-based regimens on HPV infection dynamics. Expanding HPV vaccination programs targeting MSM and PWH in Mexico, alongside routine anal HPV screening, could significantly reduce the burden of HPV-related cancers. Furthermore, integrating educational interventions to address modifiable risk factors, such as smoking and inconsistent condom use, may strengthen the prevention strategies. These efforts could inform public health policies to mitigate the disproportionate impact of HPV-related diseases in PWH.

## Figures and Tables

**Table 1:** Baseline demographic, clinical, and behavioral characteristics of 214 participants with HIV, October 2023–December 2024.

Characteristics	*n*	%	Median (IQR)
**Demographic Characteristics**			
Age (years)	-	-	30 (25–36)
*Race/Ethnicity*			
Hispanic	185	86.4	-
White	23	10.7	-
Other	6	2.8	-
*Education*			
Elementary school	3	1.4	-
High school	100	46.7	-
College	84	39.3	-
Postgraduate	27	12.6	-
**Clinical Characteristics**			
Time since HIV diagnosis (months)	-	-	18 (12–29)
CD4+ T-cell count (cells/mm^3^)	-	-	625 (431–879)
*Nadir CD4+ T-Cell Count (cells/mm^3^)*			
<200	89	41.6	-
200–500	97	45.3	-
>500	28	13.1	-
HIV-1 RNA < 40 copies/mL	187	87.4	-
ART Duration (months)	-	-	12 (6–24)
*Treatment History*			
First treatment regimen	199	93	-
ART-naïve	14	6.5	-
Optimized regimen due to previous treatment failure	1	0.5	-
*ART Regimen*			
DTG/3TC/ABC	52	24.3	-
BIC/TAF/FTC	59	27.6	-
DRV/c + TDF/FTC	47	22.0	-
DTG + TDF/FTC	11	5.2	-
DRV/c + 3TC	22	10.3	-
DOR/TDF/3TC	5	2.3	-
DTG/3TC	3	1.4	-
DRV/c + DTG + TDF/FTC	1	0.5	-
None	14	6.5	-
*ART Regimen by Pharmacological Group*			
INSTI-based	126	58.9	-
PI-based	69	32.2	-
NNRTI-based	5	2.3	-
None	14	6.5	-
**Behavioral Characteristics**			
Age at first sexual intercourse (years)	-	-	17 (15–19)
Age at first receptive anal intercourse (years)	-	-	18 (16–21)
*Sexual Partners per Year*			
0	22	10.3	-
1–4	120	56.1	-
5–9	31	14.5	-
10–19	21	9.8	-
≥20	15	7.0	-
Unknown	5	2.3	-
Consistent Condom Use	182	85.0	-
Smoking	57	26.6	-
Alcohol Consumption (past 12 months)	184	86.0	-
*HPV Vaccination*			
Not vaccinated	155	72.4	-
1 Dose	28	13.1	-
2 Doses	16	7.5	-
3 Doses	14	6.5	-

**Abbreviations:** ART: antiretroviral therapy. DTG: dolutegravir, 3TC: lamivudine, ABC: abacavir. BIC: bictegravir, TAF: tenofovir alafenamide, FTC: emtricitabine. DRV/c: darunavir/cobicistat. TDF: tenofovir disoproxil fumarate, DOR: doravirine, INSTI: integrase strand transfer inhibitor, PI: protease inhibitor, NNRTI: non-nucleoside reverse-transcriptase inhibitor, HPV: human papillomavirus.

**Table 2:** Bivariate analysis of factors associated with HPV infection in 214 participants with HIV, October 2023–December 2024.

Variable	HPV Genotype	OR	95% CI	*p*-Value
**Antiretroviral Therapy (ART)**				
No INSTI use	-	1.00	Referent	-
INSTI use	16	0.42	0.21–0.83	0.011
	33	0.39	0.13–1.11	0.071
	43	0.47	0.21–1.05	0.065
No PI use	-	1.00	Referent	-
PI use	16	2.16	1.09–4.29	0.025
	6	2.38	1.06–5.32	0.031
No ART	-	1.00	Referent	-
ART use	39	0.18	0.05–0.62	0.012
	53	0.24	0.07–0.88	0.044
	66	0.20	0.06–0.68	0.016
	40	0.17	0.04–0.73	0.035
INSTI Use < 12 months	-	1.00	Referent	-
INSTI Use > 12 months	53	0.39	0.15–0.99	0.043
**Clinical Characteristics**				
CD4+ < 500 cells/mm^3^	-	1.00	Referent	-
CD4+ ≥ 500 cells/mm^3^	High-risk genotypes	0.48	0.27–0.86	0.013
	Low-risk genotypes	0.45	0.24–0.83	0.010
CD4+ < 200 cells/mm^3^	-	1.00	Referent	-
CD4+ ≥ 200 cells/mm^3^	82	0.10	0.01–0.61	0.039
HIV-1 RNA > 40 copies/mL	-	1.00	Referent	-
HIV-1 RNA < 40 copies/mL	High-risk genotypes	0.40	0.15–0.95	0.049
	45	0.22	0.06–0.82	0.036
	53	0.22	0.08–0.58	0.001
	44	0.32	0.12–0.86	0.019
**Behavioral and Demographic Characteristics**				
Non-smoking	-	1.00	Referent	-
Smoking	53	2.64	1.11–6.31	0.024
	44	2.72	1.17–6.31	0.016
Inconsistent use	-	1.00	Referent	-
Consistent condom use	39	0.84	0.79–0.89	0.030
Higher educational level	-	1.00	Referent	-
Having a lower educational level (high school or less)	16	2.15	1.09–4.23	0.025
	53	2.75	1.16–6.54	0.018
	59	4.12	1.41–12.01	0.006
Lower educational level	-	1.00	Referent	-
Having a higher educational level (college school or higher)	53	0.19	0.05–0.66	0.004

**Abbreviations:** HPV: human papillomavirus; OR: odds ratio; CI: confidence interval; ART: antiretroviral therapy; INSTI: integrase strand transfer inhibitor; PI: protease inhibitor.

**Table 3:** Adjusted odds ratio with multivariate logistic regression for HPV 16 and 53 in people living with HIV.

HPV-16 Model (*n* = 43/214)	aOR	95% (CI)	*p*-Value
INSTI	0.43	0.21–0.86	0.018
IP	1.06	0.33–3.45	0.913
Lower educational level	2.05	1.03–4.08	<0.001
Higher educational level	2.89	0.78–10.75	0.040
**HPV-53 Model (*n* = 24/214)**	**aOR**	**95% (CI)**	***p*** **-Value**
HIV-1 RNA < 40 copies/mL	0.172	0.05–0.50	<0.001
Smoking	3.025	1.16–7.88	*0.024*
Having a lower educational level	16.810	1.33–212.47	*0.029*
Having a higher educational level	0.176	0.48–0.65	*0.009*

**Abbreviations:** HPV: human papillomavirus. OR: odds ratio; CI: confidence interval; INSTI: integrase strand transfer inhibitor, PI: protease inhibitor.

## Data Availability

The data that support the findings of this study are available on request from the corresponding author (J.A.M.-M.). The data are not publicly available due to privacy or ethical restrictions.

## Supplementary Materials

The following supporting information can be downloaded at: https://www.mdpi.com/article/10.3390/v17081147/s1, Table S1: Multivariate logistic regression analysis of associated factors and HPV genotype.

## Abbreviations

3TC	Lamivudine
ABC	Abacavir
ART	Antiretroviral therapy
BIC	Bictegravir
CI	Confidence interval
DOR	Doravirine
DRV/c	Darunavir/cobicistat
DTG	Dolutegravir
FTC	Emtricitabine
HPV	Human papillomavirus
INSTI	Integrase strand transfer Inhibitor
NNRTI	Non-nucleoside retrotranscriptase inhibitor
OR	Odds ratio
PI	Protease inhibitor
TAF	Tenofovir alafenamide
TDF	Tenofovir disoproxil fumarate
