# Supplementary material for: Prevalence and Associated Factors for HPV in People Living with HIV: Are INSTIs Protective Against HPV-16? The GAIA Study
Source: Viruses. 2025 Aug 21;17(8):1147. doi: 10.3390/v17081147 (PMC12390648; doi:10.3390/v17081147)
Supplement: Supplementary file 1 [file viruses-17-01147-s001.zip › viruses-3707688-supplementary.pdf]

Supplementary Table S1. Multivariate logistic regression analysis of associated factors and HPV genotype

HPV 16 (n=43/214)			
Variable	aOR	*p*	IC
INSTI Use	0.43	0.018	0.21-0.86
PI Use	1.06	0.913	0.33-3.45
HIV-1 RNA < 40 copies/mL (Undetectable)	1.882	0.401	0.430–8.239
INSTI Use >12 months	0.487	0.41	0.088–2.698
CD4+ ≥ 500 cells/mm³	2.123	0.127	0.808–5.577
CD4+ ≥ 200 cells/mm³	0.482	0.47	0.066–3.500
HIV-1 RNA < 40 copies/mL	1.882	0.401	0.430–8.239
Smoking	0.447	0.11	0.167–1.201
Consistent Condom Use	0.606	0.374	0.201–1.827
Having a higher educational level	2.89	0.040	0.78-10.75
Having a lower educational level	2.05	<0.001	1.03-4.08

HPV 18 (n=30/214)			
Variable	aOR	*p*	IC
INSTI Use	2.185	0.526	0.195–24.530
PI Use	2.257	0.474	0.243–20.956
INSTI Use >12 months	2.128	0.282	0.538–8.419
CD4+ ≥ 500 cells/mm³	0.603	0.226	0.266–1.367
CD4+ ≥ 200 cells/mm³	2.429	0.504	0.179–32.892
HIV-1 RNA < 40 copies/mL	1.989	0.373	0.438–9.029
Smoking	0.579	0.284	0.213–1.575
Consistent Condom Use	0.34	0.064	0.108–1.065
Having higher educational level	0.423	0.06	0.173–1.035
Having a lower educational level	2.419	0.568	0.117–50.088

HPV 31 (n=17/214)			
Variable	aOR	*p*	IC
INSTI Use	0.347	0.008	0.155–0.778
PI Use	0.826	0.87	0.096–7.099
INSTI Use >12 months	0.289	0.099	0.070–1.194
CD4+ ≥ 500 cells/mm³	1.869	0.372	0.478–7.311
CD4+ ≥ 200 cells/mm³	2.013	0.758	0.310–13.072
HIV-1 RNA < 40 copies/mL	0.686	0.695	0.105–4.479
Smoking	1.369	0.559	0.451–4.155
Consistent Condom Use	0.292	0.038	0.091–0.932
Having higher educational level	0.268	0.048	0.073–0.988
Having a lower educational level	0.714	0.808	0.042–12.190

HPV 33 (n=16/214)

Variable	aOR	p	IC
INSTI Use	0.018	0.007	0.001–0.327
PI Use	0.047	0.037	0.003–0.836
INSTI Use >12 months	1.572	0.538	0.382–6.472
CD4+ ≥ 500 cells/mm³	1.706	0.486	0.365–7.980
CD4+ ≥ 200 cells/mm³	2.249	0.469	0.228–22.191
HIV-1 RNA < 40 copies/mL	0.048	0.057	0.002–1.094
Smoking	0.84	0.74	0.273–2.592
Consistent Condom Use	0.28	0.07	0.071–1.109
Having higher educational level	12.219	0.051	0.989–151.006
Having a lower educational level	1.263	0.849	0.123–12.962

HPV 35 (n=17/214)			
Variable	aOR	p	IC
INSTI Use	1.026	0.968	0.261–4.040
PI Use	2.12	0.428	0.310–14.518
INSTI Use >12 months	0.878	0.859	0.203–3.794
CD4+ ≥ 500 cells/mm³	0.764	0.732	0.154–3.803
CD4+ ≥ 200 cells/mm³	7442573804005889.0	0.998	0.000–.
HIV-1 RNA < 40 copies/mL	1.279	0.792	0.205–7.986
Smoking	1.492	0.559	0.366–6.079
Consistent Condom Use	0.754	0.603	0.247–2.301
Having higher educational level	109153683.512	0.998	0.000–.
Having a lower educational level	170049949.05	0.998	0.000–.

HPV 39 ((n=26/214)			
Variable	aOR	p	IC
INSTI Use	0.213	0.011	0.064–0.704
PI Use	0.224	0.022	0.063–0.805
INSTI Use >12 months	0.76	0.798	0.090–6.404
CD4+ ≥ 500 cells/mm³	0.824	0.844	0.106–6.388
CD4+ ≥ 200 cells/mm³	1.596	0.758	0.058–43.825
HIV-1 RNA < 40 copies/mL	0.9	0.908	0.104–7.799
Smoking	0.959	0.95	0.183–5.019
Consistent Condom Use	0.802	0.74	0.211–3.052
Having higher educational level	0.438	0.103	0.163–1.180
Having a lower educational level	235923508.904	0.998	0.000–.

HPV 45 (n=11/214)			
Variable	aOR	p	IC
INSTI Use	0.617	0.478	0.157–2.431

PI Use	0.834	0.842	0.152–4.577
INSTI Use >12 months	0.826	0.794	0.212–3.217
CD4+ ≥ 500 cells/mm³	1.719	0.547	0.275–10.752
CD4+ ≥ 200 cells/mm³	1.165	0.931	0.080–16.897
HIV-1 RNA < 40 copies/mL	10.539	0.005	2.027–54.792
Smoking	0.824	0.772	0.200–3.402
Consistent Condom Use	0.563	0.403	0.140–2.268
Having higher educational level	0.581	0.463	0.134–2.515
Having a lower educational level	0.98	0.979	0.158–6.082

HPV 51 (n=30/214)			
Variable	aOR	*p*	IC
INSTI Use	0.892	0.915	0.169–4.699
PI Use	0.891	0.933	0.159–4.990
INSTI Use >12 months	0.981	0.985	0.190–5.062
CD4+ ≥ 500 cells/mm³	1.221	0.827	0.222–6.726
CD4+ ≥ 200 cells/mm³	1.476	0.805	0.102–21.312
HIV-1 RNA < 40 copies/mL	0.928	0.938	0.183–4.710
Smoking	0.924	0.938	0.243–3.518
Consistent Condom Use	0.8	0.723	0.232–2.753
Having higher educational level	2.617	0.032	1.085–6.312
Having a lower educational level	3.412	0.05	1.002–11.622

HPV 52 (n=15/214)			
Variable	aOR	*p*	IC
INSTI Use	0.642	0.55	0.149–2.768
PI Use	0.518	0.399	0.108–2.489
INSTI Use >12 months	0.477	0.353	0.104–2.177
CD4+ ≥ 500 cells/mm³	0.967	0.963	0.210–4.446
CD4+ ≥ 200 cells/mm³	0.872	0.939	0.081–9.389
HIV-1 RNA < 40 copies/mL	1.568	0.659	0.206–11.927
Smoking	0.959	0.938	0.221–4.158
Consistent Condom Use	0.683	0.604	0.152–3.062
Having higher educational level	0.364	0.127	0.100–1.332
Having a lower educational level	0.859	0.888	0.147–5.032

HPV 53 (n=24/214)			
Variable	aOR	*p*	IC
INSTI Use	0.993	0.993	0.214–4.600
PI Use	0.708	0.726	0.109–4.613
INSTI Use >12 months	1.043	0.954	0.203–5.366
CD4+ ≥ 500 cells/mm³	1.102	0.894	0.211–5.752

CD4+ ≥ 200 cells/mm³	1.769	0.682	0.094–33.424
HIV-1 RNA < 40 copies/mL	0.172	<0.001	0.05-0.50
Smoking	3.025	0.024	1.16-7.88
Consistent Condom Use	0.935	0.927	0.243–3.602
Having higher educational level	0.176	0.009	0.48-0.65
Having a lower educational level	16.810	0.029	1.33-212.47

HPV 56 (n=23/214)			
Variable	aOR	*p*	IC
INSTI Use	0.831	0.701	0.322–2.141
PI Use	0.34	0.263	0.052–2.245
INSTI Use >12 months	0.718	0.724	0.114–4.507
CD4+ ≥ 500 cells/mm³	0.547	0.175	0.229–1.308
CD4+ ≥ 200 cells/mm³	2.565	0.504	0.162–40.591
HIV-1 RNA < 40 copies/mL	0.378	0.232	0.076–1.865
Smoking	0.26	0.039	0.072–0.932
Consistent Condom Use	1.076	0.923	0.243–4.768
Having higher educational level	3.553	0.036	1.086–11.618
Having a lower educational level	7.175	0.077	0.807–63.777

HPV 58 (n=26/214)			
Variable	aOR	*p*	IC
INSTI Use	0.831	0.701	0.322–2.141
PI Use	0.34	0.263	0.052–2.245
INSTI Use >12 months	0.718	0.724	0.114–4.507
CD4+ ≥ 500 cells/mm³	0.547	0.175	0.229–1.308
CD4+ ≥ 200 cells/mm³	2.565	0.504	0.162–40.591
HIV-1 RNA < 40 copies/mL	0.378	0.232	0.076–1.865
Smoking	0.26	0.039	0.072–0.932
Consistent Condom Use	1.076	0.923	0.243–4.768
Having higher educational level	3.553	0.036	1.086–11.618
Having a lower educational level	2.116	0.408	0.358–12.506

HPV 59 (n=22/214)			
Variable	aOR	*p*	IC
INSTI Use	1.374	0.862	0.039–48.974
PI Use	1.471	0.686	0.226–9.567
INSTI Use >12 months	0.756	0.671	0.208–2.748
CD4+ ≥ 500 cells/mm³	1.719	0.395	0.493–5.987
CD4+ ≥ 200 cells/mm³	0.521	0.648	0.032–8.575
HIV-1 RNA < 40 copies/mL	1.897	0.456	0.353–10.206
Smoking	0.207	0.028	0.051–0.841
Consistent Condom Use	1.762	0.472	0.376–8.247

Having higher educational level	4.199	0.226	0.412–42.796
Having a lower educational level	9.198	0.001	2.450–34.537

HPV 66 (n=28/214)			
Variable	aOR	*p*	IC
INSTI Use	0.239	0.023	0.069–0.824
PI Use	0.237	0.038	0.061–0.925
INSTI Use >12 months	0.419	0.432	0.048–3.677
CD4+ ≥ 500 cells/mm³	2.238	0.13	0.788–6.355
CD4+ ≥ 200 cells/mm³	0.622	0.69	0.060–6.422
HIV-1 RNA < 40 copies/mL	1.915	0.476	0.321–11.422
Smoking	0.357	0.109	0.101–1.258
Consistent Condom Use	0.812	0.75	0.225–2.934
Having higher educational level	0.524	0.247	0.176–1.566
Having a lower educational level	0.527	0.482	0.088–3.143

HPV 68 (n=15/214)			
Variable	aOR	*p*	IC
INSTI Use	1.495	0.862	0.016–138.808
PI Use	2.073	0.213	0.658–6.527
INSTI Use >12 months	1.729	0.627	0.190–15.766
CD4+ ≥ 500 cells/mm³	1.493	0.584	0.356–6.264
CD4+ ≥ 200 cells/mm³	0.166	0.173	0.012–2.201
HIV-1 RNA < 40 copies/mL	1.901	0.512	0.279–12.965
Smoking	0.956	0.953	0.214–4.274
Consistent Condom Use	0.442	0.252	0.109–1.788
Having higher educational level	0.575	0.404	0.157–2.108
Having a lower educational level	0.552	0.608	0.057–5.370

HPV 69 (n=9/214)			
Variable	aOR	*p*	IC
INSTI Use	0.0	0.995	0.000–.
PI Use	0.0	0.992	0.000–.
INSTI Use >12 months	0.178	0.388	0.003–9.020
CD4+ ≥ 500 cells/mm³	12.735	0.223	0.212–764.526
CD4+ ≥ 200 cells/mm³	921884.996	0.999	0.000–.
HIV-1 RNA < 40 copies/mL	0.0	0.994	0.000–.
Smoking	19.024	0.267	0.105–3441.526
Consistent Condom Use	207610480823926.4	0.995	0.000–.
Having higher educational level	72919499.338	0.997	0.000–.
Having a lower educational level	81.963	0.027	1.634–4112.465

HPV 73 (n=15/214)			
Variable	aOR	*p*	IC
INSTI Use	0.0	0.998	0.000–.
PI Use	0.388	0.356	0.052–2.897
INSTI Use >12 months	0.463	0.184	0.149–1.441
CD4+ ≥ 500 cells/mm³	1.071	0.926	0.252–4.559
CD4+ ≥ 200 cells/mm³	0.848	0.91	0.047–15.139
HIV-1 RNA < 40 copies/mL	0.259	0.435	0.009–7.674
Smoking	0.542	0.451	0.110–2.662
Consistent Condom Use	1.831	0.534	0.272–12.342
Having higher educational level	0.863	0.852	0.182–4.089
Having a lower educational level	0.841	0.89	0.073–9.692

HPV 82 (n=8/214)			
Variable	aOR	*p*	IC
INSTI Use	0.0	0.998	0.000–.
PI Use	0.305	0.649	0.002–50.106
INSTI Use >12 months	0.309	0.32	0.031–3.119
CD4+ ≥ 500 cells/mm³	3.441	0.316	0.307–38.624
CD4+ ≥ 200 cells/mm³	0.067	0.013	0.008–0.560
HIV-1 RNA < 40 copies/mL	0.169	0.507	0.001–32.205
Smoking	2.496	0.529	0.144–43.195
Consistent Condom Use	0.631	0.786	0.023–17.561
Having higher educational level	0.631	0.786	0.023–17.561
Having a lower educational level	0.59	1.0	0.000–.

HPV 6 (n=28/214)			
Variable	aOR	*p*	IC
INSTI Use	5.195	0.163	0.513–52.646
PI Use	2.529	0.03	1.092–5.853
INSTI Use >12 months	0.385	0.394	0.043–3.459
CD4+ ≥ 500 cells/mm³	1.015	0.98	0.325–3.171
CD4+ ≥ 200 cells/mm³	0.18	0.096	0.024–1.353
HIV-1 RNA < 40 copies/mL	0.18	0.096	0.024–1.353
Smoking	0.639	0.433	0.208–1.959
Consistent Condom Use	0.437	0.179	0.131–1.462
Having higher educational level	1.334	0.688	0.327–5.436
Having a lower educational level	1.701	0.651	0.171–16.929

HPV 11 (n=13/214)			
Variable	aOR	*p*	IC
INSTI Use	1028223176.962	0.998	0.000–.
PI Use	1198847610.223	0.998	0.000–.
INSTI Use >12 months	0.367	0.399	0.036–3.771
CD4+ ≥ 500 cells/mm³	0.681	0.653	0.127–3.643
CD4+ ≥ 200 cells/mm³	0.043	0.1	0.001–1.832
HIV-1 RNA < 40 copies/mL	1.595	0.738	0.103–24.617
Smoking	1.926	0.345	0.494–7.506
Consistent Condom Use	2.687	0.389	0.284–25.420
Having higher educational level	1.459	0.808	0.069–30.888
Having a lower educational level	0.0	0.998	0.000–.

HPV 40 (n=13/214)			
Variable	aOR	*p*	IC
INSTI Use	0.0	0.998	0.000–.
PI Use	0.144	0.178	0.009–2.417
INSTI Use >12 months	0.0	0.998	0.000–.
CD4+ ≥ 500 cells/mm³	0.738	0.816	0.057–9.542
CD4+ ≥ 200 cells/mm³	0.292	0.493	0.009–9.866
HIV-1 RNA < 40 copies/mL	0.628	0.771	0.027–14.438
Smoking	3.453	0.161	0.611–19.510
Consistent Condom Use	337460092.264	0.998	0.000–.
Having higher educational level	2.004	0.639	0.109–36.687
Having a lower educational level	0.0	0.998	0.000–.
HPV 42 (n=26/214)			
Variable	aOR	*p*	IC
INSTI Use	0.704	0.439	0.289–1.713
PI Use	0.587	0.554	0.101–3.428
INSTI Use >12 months	0.48	0.515	0.053–4.376
CD4+ ≥ 500 cells/mm³	0.658	0.423	0.237–1.829
CD4+ ≥ 200 cells/mm³	0.246	0.064	0.055–1.087
HIV-1 RNA < 40 copies/mL	0.922	0.928	0.160–5.319
Smoking	0.777	0.649	0.263–2.301
Consistent Condom Use	1.47	0.59	0.362–5.962
Having higher educational level	0.827	0.784	0.211–3.233
Having a lower educational level	0.849	0.879	0.103–7.025

HPV 44 (n=26/214)			
Variable	aOR	*p*	IC
INSTI Use	0.261	0.279	0.023–2.964

PI Use	3.03	0.151	0.668–13.753
INSTI Use >12 months	0.667	0.373	0.273–1.628
CD4+ ≥ 500 cells/mm³	0.532	0.273	0.172–1.644
CD4+ ≥ 200 cells/mm³	0.222	0.056	0.047–1.040
HIV-1 RNA < 40 copies/mL	2.049	0.198	0.687–6.114
Smoking	2.619	0.032	1.087–6.313
Consistent Condom Use	0.541	0.328	0.158–1.853
Having higher educational level	0.827	0.784	0.211–3.233
Having a lower educational level	1.595	0.521	0.383–6.639

HPV 54 (n=11/214)			
Variable	aOR	*p*	IC
INSTI Use	1.17	0.922	.052–26.530
PI Use	3.063	0.144	.683–13.732
INSTI Use >12 months	0.667	0.373	.273–1.628
CD4+ ≥ 500 cells/mm³	0.532	0.273	.172–1.644
CD4+ ≥ 200 cells/mm³	0.222	0.056	.047–1.040
HIV-1 RNA < 40 copies/mL	2.049	0.198	.687–6.114
Smoking	2.47	0.260	0.512–11.924
Consistent Condom Use	0.541	0.328	.158–1.853
Having higher educational level	1.05	0.954	.197–5.596
Having a lower educational level	1.595	0.521	.383–6.639

HPV 61 (n=14/214)			
Variable	aOR	*p*	IC
INSTI Use	0.73	0.819	0.050–10.744
PI Use	0.171	0.093	0.022–1.346
INSTI Use >12 months	2.521	0.166	0.681–9.329
CD4+ ≥ 500 cells/mm³	2.499	0.179	0.656–9.518
CD4+ ≥ 200 cells/mm³	111514774.342	0.999	0.000–.
HIV-1 RNA < 40 copies/mL	0.845	0.921	0.031–23.228
Smoking	0.225	0.158	0.028–1.788
Consistent Condom Use	0.795	0.782	0.156–4.040
Having higher educational level	0.981	0.985	0.131–7.361
Having a lower educational level	0.967	0.979	0.078–12.036

HPV 70 (n=15/214)			
Variable	aOR	*p*	IC
INSTI Use	0.0	0.998	0.000–.

PI Use	1.66	0.782	0.046–59.989
INSTI Use >12 months	0.757	0.632	0.241–2.373
CD4+ ≥ 500 cells/mm³	1.365	0.643	0.365–5.101
CD4+ ≥ 200 cells/mm³	0.591	0.703	0.040–8.815
HIV-1 RNA < 40 copies/mL	1.521	0.656	0.240–9.634
Smoking	1.864	0.326	0.538–6.463
Consistent Condom Use	3.508	0.275	0.369–33.349
Having higher educational level	0.638	0.492	0.178–2.293
Having a lower educational level	1.074	0.939	0.171–6.739
